# Considering culture, context and community in mhGAP implementation and training: challenges and recommendations from the field

**DOI:** 10.1186/s13033-019-0312-9

**Published:** 2019-08-24

**Authors:** Neda Faregh, Raphael Lencucha, Peter Ventevogel, Benyam Worku Dubale, Laurence J. Kirmayer

**Affiliations:** 1 Department of Psychology, Carleton University, 1125 Colonel By Drive, Ottawa, ON K1S 5B6 Canada; 2 School of Physical & Occupational Therapy, McGill University, 3654 Prom Sir-William-Osler, Montreal, QC H3G 1Y5 Canada; 3 Public Health Section, Division of Programme Support and Management, United Nations High Commissioner for Refugees, 94 Rue de Montbrillant, 1202 Geneva, Switzerland; 4 Department of Psychiatry, School of Medicine, College of Health Sciences, Addis Ababa University, Addis Ababa, Ethiopia; 5 Division of Social and Transcultural Psychiatry, McGill University, 1033 Pine Ave, Montreal, QC H3A 1A1 Canada; 6 Global Mental Health Program, McGill University, Montreal, Canada

**Keywords:** mhGAP, Global Mental Health, Primary care, Integration, Task shifting, Cultural adaptation, Implementation

## Abstract

**Background:**

Major efforts are underway to improve access to mental health care in low- and middle-income countries (LMIC) including systematic training of non-specialized health professionals and other care providers to identify and help individuals with mental disorders. In many LMIC, this effort is guided by the mental health Gap Action Programme (mhGAP) established by the World Health Organization, and commonly centres around one tool in this program: the mhGAP-Intervention Guide.

**Objective:**

To identify cultural and contextual challenges in mhGAP training and implementation and potential strategies for mitigation.

**Method:**

An informal consultative approach was used to analyze the authors’ combined field experience in the practice of mhGAP implementation and training. We employed iterative thematic analysis to consolidate and refine lessons, challenges and recommendations through multiple drafts. Findings were organized into categories according to specific challenges, lessons learned and recommendations for future practice. We aimed to identify cross-cutting and recurrent issues.

**Results:**

Based on intensive fieldwork experience with a focus on capacity building, we identify six major sets of challenges: (i) cultural differences in explanations of and attitudes toward mental disorder; (ii) the structure of the local health-care system; (iii) the level of supervision and support available post-training; (iv) the level of previous education, knowledge and skills of trainees; (v) the process of recruitment of trainees; and (vi) the larger socio-political context. Approaches to addressing these problems include: (1) cultural and contextual adaptation of training activities, (2) meaningful stakeholder and community engagement, and (3) processes that provide support to trainees, such as ongoing supervision and Communities of Practice.

**Conclusion:**

Contextual and cultural factors present major barriers to mhGAP implementation and sustainability of improved services. To enable trainees to effectively apply their local cultural knowledge, mhGAP training needs to: (1) address assumptions, biases and stigma associated with mental health symptoms and problems; (2) provide an explicit framework to guide the integration of cultural knowledge into assessment, treatment negotiation, and delivery; and (3) address the specific kinds of problems, modes of clinical presentations and social predicaments seen in the local population. Continued research is needed to assess the effectiveness these strategies.

## Background

Despite increasing recognition of the high global burden of mental, neurological and substance use disorders [1, 2] and the development of low-cost and effective treatments for some of these disorders, most people in low- and middle-income countries (LMIC)^1^ have limited access to mental health services [3]. Rates of treatment in LMIC range from 5 to 13% of people with mental disorders [4]. This discrepancy has been framed as a treatment gap [5] and can be due to insufficient and inequitable distribution of resources as well as inefficient use of existing services [6]. At the system level, the response to this gap is affected by political will and the often low priority given to mental health in the health-care system [7, 8]. At the community level, cultural beliefs and stigma associated with mental disorders shape help-seeking behaviors [9–13]. Mental health service delivery warrants special attention because deficiencies in this area are associated with great suffering, morbidity and mortality, economic costs, and human rights abuses for those afflicted by mental disorders and their families [14]. Strengthening mental health services in LMIC has been identified as a global health priority [15].

In an effort to respond to this priority, the World Health Organization (WHO) has developed a set of tools, including the mhGAP Intervention Guide (mhGAP-IG) [16, 17] along with guidelines, recommended strategies and related reference documents for training and implementation [18]. The priority conditions addressed by mhGAP-IG include: depression, psychosis (including schizophrenia), bipolar disorders, epilepsy, developmental and behavioral disorders in children and adolescents, dementia, alcohol use disorders, drug use disorders, self-harm/suicide and other significant emotional or medically unexplained complaints. The mhGAP Intervention Guide was developed as a clinical tool and is often used in training and capacity building with non-specialist service providers. At present, there is no comprehensive repository of mhGAP training implementation initiatives or programs. However, peer-reviewed, and grey literature [19–21], international aid agency and NGO reports indicate that mhGAP training is now the most widely used mental health capacity building tool in LMIC [22–27]. A recent systematic review found that mhGAP tools had been used in 90 countries [22]. Some projects used the full package while others chose to work with one or more modules. Despite its widespread adoption, the implementation process of mhGAP remains largely untested. An important facet of mhGAP implementation is its utility in capacity building. A few groups have been engaged in documenting the process of adaptation, implementation, and evaluating training outcomes. Examples include the Programme for Improving Mental health Care (PRIME) [28], and EMERALD: Emerging mental health systems in low and middle-income countries [29] projects, and the interventional study from Ethiopia involved in training and education of primary care health professionals in mhGAP [30]. The most effective ways to utilize the increased capacity of mhGAP trained providers within primary care are not yet well demonstrated. However, the need to attend to the factors that shape mhGAP implementation and evaluation is widely recognized [31].

In this article, we draw from our field experience to discuss challenges encountered in the process of mhGAP training and implementation and provide recommendations for future work. The fieldwork informing our reflections include the authors’ individual experiences leading, organizing, delivering, and evaluating mhGAP implementation projects in multiple countries and settings including Chad, Ethiopia, Nigeria, Guinea, and Haiti, over a period of 6 years. We have consolidated, organized, compared, and critically analyzed these experiences in order to inform future dialogue, research and practice. Our approach to the analysis discussed in more detail in “Method” section.

Our experience suggests that successful mhGAP implementation and capacity building requires not merely didactic and practical training but also broader implementation of support systems and interventions that are responsive to local needs and cultural contexts. The lessons learned about mhGAP implementation can inform future research and practice and assist those involved with mhGAP program implementation at the levels of health policy, training, community and health clinic.

## The goals and structure of mhGAP

The mhGAP training tools are designed for use with non-specialized health service providers from a wide variety of backgrounds. In practice, the participants in training may include physicians, nurses, midwives, health technicians, community health workers (CHW) and, in rare instances, traditional healers. Although CHW and traditional healers are not the intended audience of mhGAP training they are sometimes included in training programs [26, 32–34]. The goal of these training tools is to increase primary mental health care in LMIC using a task-shifting approach. Task-shifting refers to the practice of redistributing some tasks from highly qualified health workers to health workers with fewer qualifications and shorter training [35]. The WHO’s global guidelines for task-shifting recommend this approach as a method of expanding healthcare delivery workforce by increasing the total number of health workers. The mhGAP training tools are freely accessible and available in multiple languages including Arabic, English, French, Japanese, Marathi, Persian, Portuguese, Russian and Spanish [36]. The training manuals recommend a 1-week training course that includes didactic modules in addition to instructional videos, group work, and role-plays. The documents are intended to be adapted to the context of each setting.

The mhGAP program emphasizes clinical decision-making algorithms and stepped-care treatment protocols to assess and manage a set of priority mental disorders. These manuals have been adapted for multiple settings including refugee and humanitarian crises in the mhGAP-Humanitarian Intervention Guide (mhGAP-HIG) [37]. The mhGAP-HIG is more concise, with several modules left out and new modules added on topics relevant to humanitarian settings including acute stress, grief, and posttraumatic stress disorder (PTSD) [38]. Following the first publication of the mhGAP-IG in 2010, the mhGAP-IG 2.0 was released in 2016 and the WHO plans to provide regular guideline updates every 2 years and new releases every 5 years.

Throughout this text we use the term mhGAP to refer to the process of mhGAP-IG and mhGAP-HIG training and the implementation of mhGAP program. We use the term trainee to refer to any learner attending mhGAP-IG or mhGAP-HIG training.

## Challenges in implementing mhGAP training programs in LMIC

A key concern of mhGAP implementation is how trainees apply their knowledge in practice. Ensuring that mhGAP training results in improved services depends in part on understanding the structural and systemic factors that influence service provision post-training. Implementation requires effective leadership, ongoing training in early implementation phases, and ensuring retention of trained providers [39]. There are multiple challenges inherent in implementing the complex program of training, organizational changes involved in task shifting, and ongoing mentorship and activities needed for sustainability in community settings.

The mhGAP program is based on a biomedical model with particular assumptions about the nature of mental health problems. The program is agnostic to health-care providers’ own ethnocultural backgrounds and interpretations. However, it implicitly endorses the framework of biomedical psychiatric and individualistic psychology in its approach to mental health and illness in terms of discrete problems located within the individual [40–42]. There is a substantial body of research highlighting the importance of culturally informed pedagogy and culturally sensitive and responsive mental health programs that build on the experiences and knowledge of communities. However, there is little work examining the situated experiences of mhGAP trainers and program implementers and how the interactions of social, contextual and cultural factors may impact on the implementation process and outcome. Given the limited literature addressing field experience related to mhGAP, we aimed to identify key themes and issues from our experiences.

## Method

This article presents systematic reflections and post hoc analysis of the authors’ informal observations during diverse field experiences in mhGAP-related projects at different sites. The projects were carried out in multiple areas in six countries (Chad, Ethiopia, Nigeria, Guinea, and Haiti) and included distance training. We did not collect or use any participant or personal data and hence no ethical approval for the project was needed. All references to data related to these projects are based on published articles and cited when relevant. Findings were organized into categories according to specific challenges, lessons learned and recommendations for future practice. We aimed to identify cross-cutting and recurrent issues. Given this goal, and to maintain confidentiality, we do not identify specific sources.

### Approach

We used an informal consultative approach to the analysis of our combined field experience in the practice of mhGAP implementation and training. The accumulated expertise were based on individual work and familiarity with project reports and memos, stories of experiences, participant observation, field-notes, ongoing conversations with key stakeholders and peers, academic presentations where critical reflections were provided, as well as written exchanges between the authors during and after fieldwork. We also included material drawn from inquiries and requests for consultation addressed to subject-matter experts regarding issues encountered in the field, the sharing of evaluative data collected in the field as part of auxiliary programs. All of this information was organized into categories according to challenges, lessons learned and recommendations for future practice.

**Fig. 1:**
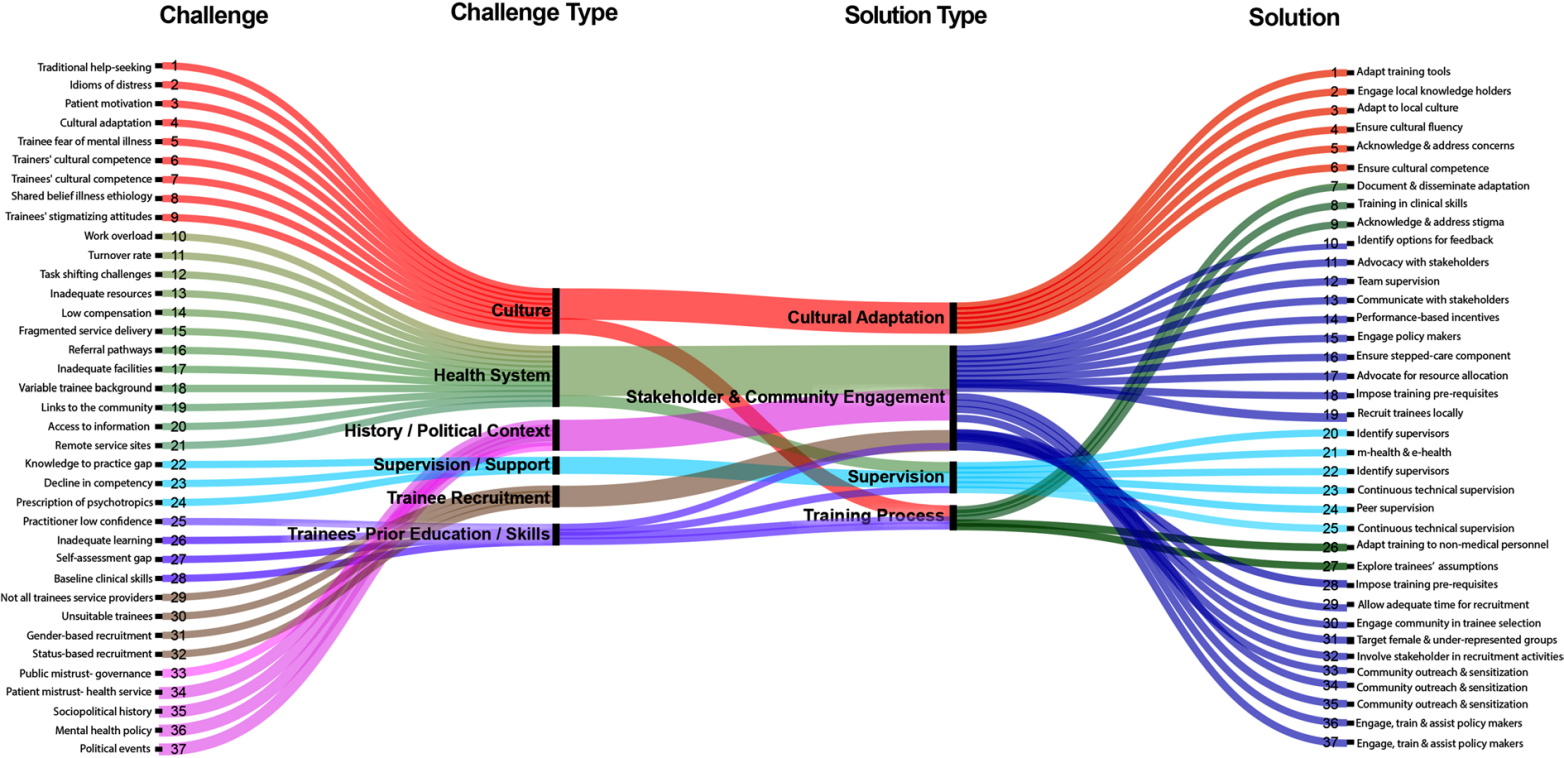
Correspondence of type of challenges in mhGAP training and implementation and potential solutions

We employed iterative thematic analysis to consolidate and refine lessons, challenges and recommendations through multiple drafts. We generated key themes and grouped each challenge and recommendation with its corresponding theme. The challenges items and the recommendations were tabulated against the themes. Microsoft Excel was used to recode items and corresponding themes from string to categorical indicator variables. These codes were used to produce an alluvial diagram using the open source Web application RawGraphs (see Fig. 1).

## Findings and discussion

We identified six sets of cultural/contextual issues that require consideration in mhGAP implementation: (i) cultural differences in explanations of and attitudes toward mental disorders; (ii) the structure of the local health-care system; (iii) the level of supervision and support available post-training; (iv) the level of previous education, knowledge and skills of trainees; (v) the process of recruitment of trainees; and (vi) the larger socio-political context of the region.

### Cultural differences in explanations of and attitudes toward mental disorders

Cultural knowledge, attitudes and practices exert strong effects on help-seeking, treatment referral, adherence, and response to interventions. Although trainees with local cultural knowledge are an essential resource in mental health service delivery, they present specific challenges to standardized mhGAP training: (1) trainees may share cultural beliefs, and assumptions with others in their local culture that lead to biases and stigma toward mental illness; (2) trainees may be unclear how to apply their cultural knowledge to the specific tasks of mhGAP; and (3) the local context may include particular types of clinical problems, presentations, and social predicaments not explicitly addressed in mhGAP.

Cultural attitudes toward mental disorders are important factors in service provision. Stigmatizing cultural beliefs, explanatory models and attitudes shared by both patients and mental health workers (e.g., the concern that mental disorders are contagious or involve supernatural causes that cannot be addressed by biomedicine) will shape service delivery. Lack of attention to cultural context on the part of providers and decision-makers can lead to mistrust of mental health information and services and reduce motivation to engage with mental healthcare or adhere to treatment [43, 44]. In our experience, it is not uncommon to meet health practitioners who are convinced that mental disorder in a specific patient is due to curses, spirit possession, or to patients having behaved in ways that angered the ancestors. Some health providers who accepted the premise that psychotic symptoms had supernatural causes, believed this could confer immunity to physical illnesses. Such views among health care providers may affect interactions with patients and influence treatment choices.

Of course, the impact of cultural beliefs is not confined to mental conditions. Given that physical illnesses often provide signs that point to the presence of known biological agents, biomedical services may be more likely to be sought for physical conditions, but cultural meanings and implications of affliction remain a critical ingredient in the negotiation of care [45]. In the absence of visible signs of disease, as is usually the case for mental disorder, there may be greater uncertainty about the nature of the affliction and its causes. A recent study in Uganda evaluating challenges to implementing the WHO guidelines for management of stress found that health care providers held that the psychological interventions developed in high-income countries were not adaptable to local settings in part because the guidelines did not address the cultural context [46].

Cultural and contextual factors also influence the types of mental health problems that practitioners face. This may pose challenges to the applicability of mhGAP materials. For example, in the Ugandan studies, practitioners found the guidelines did not provide adequate tools for the treatment of comorbid disorders and did not address the types of psychosocial problems seen in the population including war-related and residual post-conflict difficulties of Ugandan population and the psychosocial difficulties particular to refugee populations, including, for example, those related to domestic violence and disputes over land [47].

In our field work, trainees in other regions have expressed similar concerns. Examples included reported difficulty in managing cases of severe trauma, sexual violence, and child-marriages. Trainees working with refugee populations frequently expressed the need for specific training in inter-ethnic and conjugal conflict resolution. Trainees gave examples of instances they were called on to intervene in cases related to clashes among refugees, and inter-ethnic clashes between refugees and members of their host community; the latter often due to the perception that refugees were responsible for the use and depletion of common community resources such as water and fuel wood. Less common but equally challenging concerns were related to negative attitudes toward refugees held by some trainees themselves.

A large body of work demonstrates that cultural variations in illness experience and behavior influence the diagnosis and treatment of mental health problems [48, 49]. Cultural knowledge, attitudes and practices exert strong effects on help-seeking, treatment referral, adherence, and response to interventions. People from many ethnocultural backgrounds do not seek mental health-care, either because they do not conceptualize their problems as appropriate for clinical attention or because they fear social stigma [50]. Even when they do present for help, patients may receive incorrect diagnoses and inappropriate or inadequate help from care providers unfamiliar with their language, cultural background, or social situation [51]. In the context of mhGAP, this may occur if generic methods are applied without adequate cultural adaptation and contextualization. While minimal adaptation is recommended to maintain fidelity to the evidence-based protocols and not compromise their efficacy, cultural and contextual adaption are necessary to account for local idioms of distress and patterns of help seeking behavior and to provide accurate tools for case-identification and referral to more specialized care [52–54]. Crucially, cultural idioms of distress which provide everyday languages of suffering, usually cannot be simply mapped onto specific disorders but need to be explored in context case-by-case to determine the nature of the patient’s concerns [55].

When mhGAP training is carried out in humanitarian settings, it is common for trainers and trainees to come from different cultures. In Chad, for example, mental health training has been provided by trainers from Canada, Democratic Republic of Congo, India, Belgium and the US. The trainees are French-speaking Chadian health-providers who treat Zaghawa-speaking Darfur refugees. In these settings, the historical, geopolitical, and social contexts play important roles in communication that may affect training. Local trainees may have much relevant knowledge about idioms and explanatory models but lack ways to integrate this into mhGAP delivery.

As discussed earlier, mhGAP training is based on a biomedical model and aims to be agnostic to health-care providers’ own ethnocultural backgrounds and assumptions [40]. The biomedical model locates mental health problems within individuals and tends to downplay or discount social, moral, and spiritual explanations of distress [56]. However, many local mhGAP-trained health-care providers may share the cultural models of their patients. Moreover, in primary care or community settings, mhGAP trainees often are called on to treat patients with ambiguous, vague or diffuse symptoms, which may be attributed to many causes [57]. Trainees may have difficulty conceptualizing, diagnosing, and treating symptoms which suggest no clear etiology or are not grouped together with other symptoms in locally recognized syndromes.

### The structure of the local health-care system

To be useful mhGAP training must fit the structure of the local health care system and the contexts of practice. Some studies have highlighted the importance of such structural factors in mhGAP implementation. For example, an evaluation of implementing mhGAP guidelines in Uganda [47] cited the low ratio of provider to patients, lack of qualified staff, insufficient funding, lack of incentives for practitioners to modify their practice, lack of time for the addition of mental health evaluation to existing clinical work and a high level of ethnocultural diversity in patient populations as underlying barriers to adaptation and adoption.

In our work, we have observed structural challenges related to the logistics of service provision of two types: those related to tangible resources and those related to information resources. Both of these were frequently reported by trainees as obstacles to the integration of mhGAP training in their routine practice across settings including refugee camps, rural and urban health-care centers. Problems related to tangible resources included the availability of an adequate supply of psychiatric medications, and the availability of appropriate physical spaces for mental health consultations. Trainees working in very low resource settings were particularly concerned that the adoption of mhGAP could further burden the over-extended system without providing support or incentives for the increased workload.

Information resource challenges were associated with problems in communication and access. After training, most trainees received no feedback about the integration of mhGAP in the larger health care system or the specific impact of their work. Trainees frequently voiced concerns about a lack of information on how to access supervisory resources and referral pathways.

As previously mentioned, two essential components of mhGAP implementation are task shifting and the stepped care approach. These both depend on the structure of the health care system. The stepped-care approach depends on having appropriate referral pathways. Even if referral pathways are formally present, they may be infrequently used and function poorly in practice. Related to this, due to lack of a broader vision and strategy for mental health service systems in most LMIC, efforts to integrate mental health may be confined to Primary Health Centers (PHC). For example, in Ethiopia, health-care providers in local and regional hospitals were not given the requisite training to enable them to supervise PHC workers or handle patients who come through the referral system. An exclusive focus on one sector like primary care can contribute to fragmentation in the health-care system, with mental health-care sequestered in PHC and specialty mental health-care facilities found only in the larger cities, with little or no attention given to health-care providers in between, making it difficult to implement the stepped-care component of the mhGAP.

Other structural challenges that can impede efforts to implement mhGAP are related to the lack of mental health policy and planning. About half of the countries in Africa do not have mental health policy, and among those that do, nearly 40% have not updated or modified their policies since 1990 [37]. For example, in Kenya, the lack of comprehensive mental health policy has impacted on the ability to coordinate, evaluate and standardize service provision within the mental health system [58]. In Chad, our experience suggests that, while the government recognizes the need for mental health training for health service providers throughout the country, this responsibility is largely directed by international humanitarian agencies who have mainly focused on the refugee population. Another common situation is that efforts in scaling-up mental health services emerge as a reaction to dramatic health events or crises like epidemics or natural catastrophes [59–61]. This is important because mhGAP training programs are best implemented within a wider national mental health plan and policy and a cohesive mental health system.

### The level of supervision and support available after training

In our field experience, mhGAP training without follow-up supervision generally has been insufficient to ensure the integration of mental health knowledge into practice. Many difficulties in the application of mhGAP are related to errors in differential diagnosis leading to inappropriate care. In most cases, pre-post tests indicate increases in mental health knowledge with mhGAP training and, in some instances, post-training evaluations indicated that most trainees had high levels of confidence in their ability to administer mental health-care services. However, there is some indication from the same study that high levels of confidence may not correlate with post-training knowledge test scores [62]. In training sessions in which we participated, many practitioners focused on detecting psychotic symptoms and gave a diagnosis of psychosis without sufficient evidence. In other instances, trainees had difficulty in assessing symptoms in children and the elderly. Examples include trainees giving a diagnosis of psychosis to a child with developmental delays and to an elderly man with symptoms of dementia. Non-psychotic behaviors such as aggression, agitation, motor restlessness, and wandering may be routinely attributed to psychosis. Lack of experience in clinical interviewing and evaluation and insufficient history-taking may contribute to potential misdiagnosis.

In addition to the challenges of misdiagnosis, with over- or under-recognition of specific conditions, lack of supervision can also affect the kind and quality of interventions practitioners provide. In our experience in Ethiopia (mhGAP training 2015–2016), in the absence of adequate supervision, primary health-care physicians may be reluctant to prescribe medications in adequate doses because of concern about side effects. Diagnostic assessment and treatment approaches are mutually reinforcing. In the absence of supervision, health workers may attempt to fit symptoms they observe into the few broad categories of mental disorder they have learned about in the mhGAP program. This may result in over-diagnosis of some illnesses and potential over-prescription, thereby exposing patients at least to unnecessary expense and medication side-effects [63]. Under-diagnosis and under-treatment can also be observed, when the skills that have been taught in the classroom do not sufficiently generalize to routine clinical practice. More research and evidence are needed to understand the factors that support the effective translation of mhGAP training into practice at the primary care level.

### The level of previous education, knowledge and skills of trainees

As discussed above, mhGAP aims to narrow the service gap by task shifting (i.e. having primary-care providers offer mental health-care). Mental health training of healthcare providers varies considerably across countries and regions [64]. There is some evidence that in LMIC, mhGAP-trained health providers without prior mental health training or those with low levels of formal education can successfully assess patient needs and provide support when using locally validated measures [65]. However, trainee variation in education and skill levels can pose challenges for mhGAP training as the training program assumes a level of general knowledge that may not be consistently present among workers [66]. Insufficient basic health education may leave trainees with biases and misinformation about mental disorder. Lack of adequate knowledge and training is a common deterrent for mental health service providers in LMIC as they do not feel competent to care for people diagnosed with mental disorders [66]. Although mhGAP aims to reduce common assumptions that mental disorders are contagious or caused by witchcraft, it may not mitigate stigma. Some workers may harbor negative attitudes toward mental disorders that remain unchanged by their exposure to mhGAP training. We have observed that some trainees maintain the lay belief that mental disorder is contagious because they misidentify infectious diseases. For example, when asked about the origin of this notion of contagion, a nurse trainee recounted the case-history of a patient with meningitis.

Delivery of mhGAP training includes pre- and post-tests of mental health knowledge. These tests are used in almost all mhGAP training courses including refresher and train-the-trainer initiatives. Ideally these tests could provide important feedback on the success of training. However, to our knowledge, the test questions have not gone through an evidence-based process of item construction and the pre- and post-tests are not standardized measurement instruments. Their cross-cultural validity has not been established. We have observed trainees with different educational professional backgrounds and skill-levels have difficulty with some of the assessment questions. While there is an overall trend toward improvement at post-test [62], some trainees actually scored lower after the completion of the training compared to the start of the training. This may reflect trainees’ new-found appreciation for the subtleties of clinical work.

### Recruitment of trainees

Much preparation is needed prior to implementation of mhGAP training. A key logistical element that is not always considered systematically involves choosing and inviting trainees to enroll in the mhGAP training program. The process of selecting trainees is crucial to the ultimate success of mhGAP training. In our experience, relegating the task of trainee selection to beneficiary organizations or the state, may not lead to the best choice of candidates for mental health training, or the best outcomes in terms of accessible mental health-care for the underserved populations.

At the level of the clinic, we have observed that mhGAP trainees may be selected based on status and standing in their respective organizations. In some instances, this may even result in health administrators being recruited to the exclusion of clinical staff. While health administrators can certainly benefit from mhGAP training and can play a role in future training and supervision of clinical staff, their increased competence may not translate into better patient care because administrators are usually released from clinical duties. Status-based selection is also often tied to gender and cultural defined norms. Thus, female nurses, midwives, and birth attendants may be overlooked in the selection process in localities where women have lower standing in the work place or the community.

At the grassroots level, CHWs are recruited and trained as peer-support workers, sometimes without clear criteria for inclusion. This group represents an important human resource in LMIC, but recruitment strategies may ignore existing help-seeking pathways, traditional healers, and other sources of care that can hinder or promote mental health interventions. The most effective CHWs may be those who exert the most influence in their communities, who are respected opinion leaders, and who are centrally positioned in the community and able to provide information and assistance to as large a group of people as possible.

### Historical and current political context

The political context, both historical and current, of the community, region, and nation where mhGAP training is undertaken influences local health systems and the population’s willingness to adopt new health practices. In West Africa, for example, histories of political conflict and violence eroded trust in government institutions, including health care, with devastating effects on the response to the Ebola epidemic [67, 68]. Public health efforts directed at the epidemic in West Africa were met with reticence and resistance from the population in most affected regions but especially in Guinea a “crisis in confidence” [69] resulted in great resistance to public health efforts compared to that of its neighbors (Sierra Leone and Liberia) [70, 71]. The public’s fear of government systems also affected efforts at mental health system implementation and strengthening, including mhGAP training and service delivery, making it difficult to respond to mental health needs even when services were available. The propagation of misinformation, and incidents of violence directed to heath workers reflected entrenched fears of public officials, including health care workers [72]. Perhaps as a result, the service rooms of the clinics of Fraternité Medical Guinea (FMG), Guinea’s only health clinics offering mental health services by mhGAP trained physicians, remained largely empty. In what incident we observed, after mhGAP training, healthcare workers’ attempt to refer an Ebola survivor with severe mental disorder [73] for admission to the Donka hospital in Conakry, which houses the only public psychiatric service unit in the country was fraught with difficulties borne of the public’s deep-rooted distrust of government and authorities. Challenges^2^ included the family’s reluctance to consent to the patient’s medical transport to the hospital, an attack by a mob that tried to torch the inter-city ambulance transporting the patient, and the patient’s multiple attempts to flee the hospital he distrusted.

Although these were extraordinary circumstances, issues of trust and engagement with government or NGO programs are common in LMIC with histories of colonization, political instability and violence. There has been little discussion of this type of historically rooted challenge in the literature on public health efforts and mhGAP program implementation. However, these contextual factors play an important and largely implicit role in the adoption of programs such as mhGAP, both in the response to training, and in its integration in service provision. Strategies are needed to explore and address historically rooted political issues that may impede program implementation.

## Recommendations

The kinds of challenges we have outlined will vary widely across contexts with local culture, health care system structure and politics. Hence, generic strategies to address or mitigate these challenges will be context-dependent and must be locally adapted. The following broad recommendations are derived from our experiences with the challenges discussed in this paper. Each strategy requires careful consideration to determine its potential suitability and fit in a specific context or setting.

Given that mhGAP trainees are embedded in local cultures and health infrastructures, they are well positioned to carry out cultural and contextual adaptation. To do this, they need appropriate guidance to utilize their knowledge of local contexts and culture in the conceptualization and application of their knowledge to adaptation of the mhGAP tools. There are existing guidelines for tailoring clinical assessments to local contexts and methods for cultural adaptation [74, 75]. Methods for cultural and contextual adaptation and rapid development of culturally valid mental health measures are available that can be applied across settings [52, 76, 77].

While mhGAP training materials emphasize inclusion of cultural beliefs, practices, language, and social norms, in practice this step may be carried out only partially or hastily, and the collected information may not always inform practice [78]. Research indicates that more work is needed to integrate methods for effective intercultural clinical work in primary care [79]. Ethnocultural matching of health-care provider and patient can be a convenient approach to intercultural work, but cultural fluency is not equivalent to cultural competency; trainees need conceptual frameworks and practical strategies for integrating cultural knowledge into better assessment and intervention. Generic cultural competence consists of a distinct set of skills that go beyond knowledge of a particular culture [80]. A basic approach to clinical cultural assessment has been integrated into DSM-5 in the Cultural Formulation Interview (CFI) [81]. The CFI provides a useful starting point to provide mhGAP trainees with simple ways to explore key cultural and contextual factors with patients. The CFI explores the patients’ perception of social context, including family, community and wider social network, drawing out cultural conceptions of causes and consequences of symptoms and affliction, to clarify the meanings of the problem for the patient and their family or entourage. The CFI also explores cultural identity, coping, and help-seeking, including cultural factors that may affect help-seeking behaviors and clinician-patient relationship [82]. The CFI was developed and tested in international contexts [83] and can be locally adapted to address issues of specific importance to mhGAP implementation [84]. However, case formulation with information collected through the CFI may require a high level of skill and frequently encountered issues need to be built into local cultural adaptations of mhGAP.

Local cultural and contextual adaptation should be one of the core principles of mhGAP training and implementation, rather than an afterthought. This can be facilitated by establishing ways to gather feedback from health-care providers [85], patients and their families to identify strategies used by trainees to evaluate, diagnose, and treat individuals with mental distress, common challenges to service provision, and local resources available to support service delivery. The process of adaptation requires attention to both cultural and contextual components of assessment, formulation and intervention, including the ways that trainees currently recognize, explain, and manage common idioms of distress. Taking the time to gather this information will clarify the existing methods used by trainees for case formulation, local variations in illness experience, and modes of expressing distress that can inform subsequent training and improve the uptake, fit, and sustainability of mhGAP in local settings [86].

Although mhGAP holds great promise to narrow the Global Mental Health service gap, there is a critical need to determine if and when it leads to lasting changes in practitioner competencies and whether these competencies are translated into actual service delivery. There is also a need to develop educational and supervisory approaches to ensure that the trainees receive adequate support post-training. A study conducted in Nigeria with mhGAP trainees found that although diagnosis and referral improved greatly immediately following training, performance declined at 9-month follow-up [25]. This and other studies suggest that ongoing mentorship, supervision and support is needed to integrate mental health practice into primary care [87, 88].

Strategy of the Communities of Practice (CoP) may offer a viable approach to providing post-training educational and supervisory support to ensure that the trainees can apply their knowledge and skills. A CoP is a learning community that organizes itself around a shared interest with the objective of facilitating peer-interaction and reciprocated support in problem-solving and collaborative learning [89, 90]. Members can freely discuss situations and needs, explore ideas, and expand insights or perspectives. CoPs can play a useful role in cross-cultural and multi-ethnic settings by promoting engagement and collaboration among diverse practitioners [91]. They may be particularly advantageous where sustained professional-development activities are not available, as is often the case in LMIC. Where there is adequate access to technology, online CoPs can be both feasible and effective for health-care learning [92].

Community and stakeholder engagement have long been identified as key components of public health program implementation and sustainability [93, 94]. The WHO established guidelines for health program implementation and frameworks for community engagement and these were revised in light of the lessons learned during the international response to the Ebola epidemic in West Africa [95]. The mhGAP has successfully incorporated forward-thinking approaches to stakeholder collaboration and community engagement since its initiation. Program implementers and trainers are mandated to establish links with policymakers and to negotiate agreements with local authorities at all levels. Accordingly, appropriate mechanisms of stakeholder and community engagement need to be locally determined. However, existing mhGAP implementation strategies provide convenient opportunities for meaningful interactions with stakeholders and for collaborative decision making.

The recommendations pertaining to specific challenges are summarized in Table 1. A visual map of the identified themes and corresponding potential solutions is presented in Fig. 1. The summary table provides an overview of the major challenges we have observed and documented. Items are categorized into types of challenge and are matched with corresponding types of solution at the levels of cultural adaption, stakeholder and community engagement, supervision and the training process. As shown in Fig. 1, most challenges are related to existing health system structures followed by cross-cultural issues. The majority of mitigation strategies are embedded in community engagement. While not an exhaustive list, the visual mapping and the summary table represent a way to organize the challenges mhGAP programmers are likely to encounter and suggest mitigation strategies.

**Table 1:**
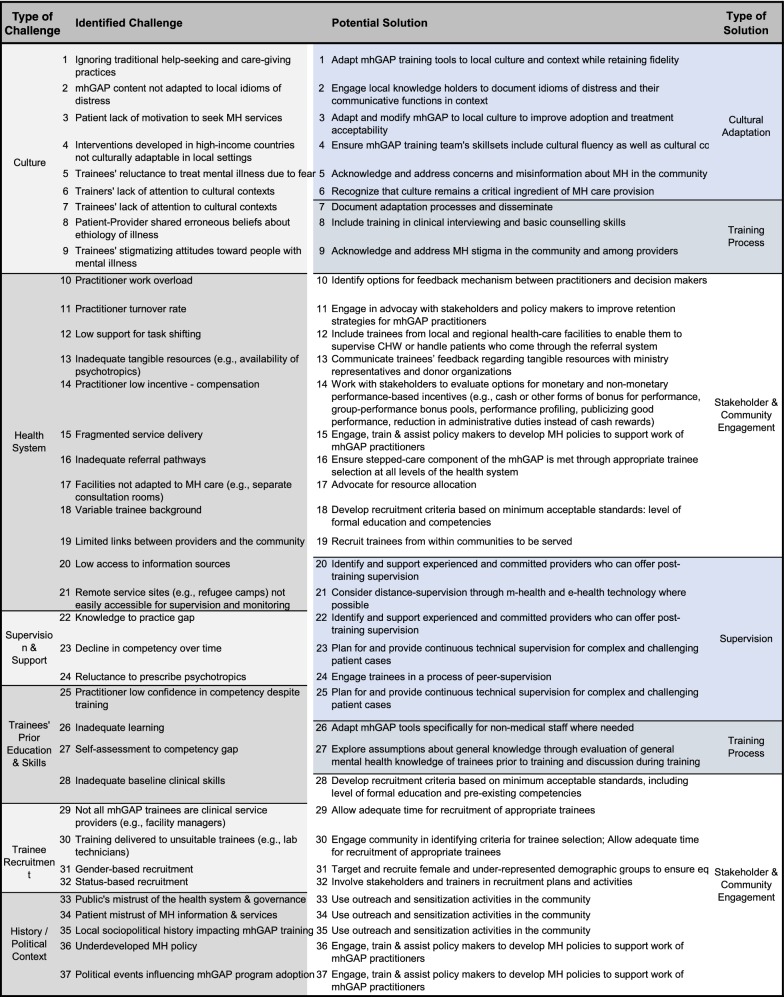
Summary of cultural and contextual challenges in mhGAP training and implementation with corresponding strategies for mitigation

Some of the suggested solutions are repeated because the same solution applies to more than one challenge

## Conclusion

Factors that can facilitate or impede mental health program implementation vary across contexts and cultures and must be examined and addressed prior to, during, and after the process of implementation. Mental health training alone cannot guarantee desired impacts on health systems and population health. Global Mental Health practice needs to remain open to new strategies and innovation in policy development, training and implementation that respect culture and context, including methods of leveraging existing resources for effective service provision, and processes that can ensure sustainability. Understanding local contexts and cultures and how they shape help-seeking and service provision requires the participation of many stakeholders, recognition of their ideas, needs, and concerns, and integration of local strategies in program adaptation, training and implementation. Incorporating culture and context and ongoing supervision and support are essential for program acceptability, effectiveness and sustainability.

To enable trainees to effectively apply their local cultural knowledge, mhGAP training needs to: (1) address any unwarranted assumptions, biases and stigma associated with mental health symptoms and problems; (2) provide an explicit framework for how to integrate cultural knowledge into assessment, treatment negotiation, and delivery; and (3) address the specific kinds of problems, modes of clinical presentations and social predicaments seen in the local population.

In sum, approaches that include deliberate cultural adaptation, contextual adaptation of training activities, meaningful stakeholder and community engagement, and processes that support the trainees such as supervision and CoPs are likely to be among the most feasible and effective methods of improving mental health integration at the primary care level in LMIC. Many of the challenges to mhGAP implementation and potential mitigation strategies discussed in this paper need further study with mixed-methods to guide the best use of resources for impact and sustainability.

## Data Availability

Not applicable.

## Abbreviations

CFI: Cultural Formulation Interview
FMG: Fraternité Medical Guinea
LMIC: low- and middle-income countries
mhGAP: mental health Gap Action Programme
mhGAP-HIG: mhGAP-Humanitarian Intervention Guide
PTSD: post traumatic stress disorder
PHC: Primary Health Centers

## Footnotes

1 The LMIC designation refers to countries’ income grouping based on the World Bank’s classification of country economies (low, lower-middle, upper-middle, and high) established by the World Bank Atlas Method from gross national income per capita estimates. For a full description of the methodology see https://datahelpdesk.worldbank.org/knowledgebase/topics/19280-country-classification.

2 We have protected individual anonymity in these observations. Aspects of the information presented were covered by the local media and is in the public domain.
